# The same growth pattern from puberty suggests that modern human diversity results from changes during pre-pubertal development

**DOI:** 10.1038/s41598-021-84327-1

**Published:** 2021-03-01

**Authors:** Jean-Claude Pineau, Fernando V. Ramirez Rozzi

**Affiliations:** 1 BABEL, UMR 8045, CNRS, UP, 2 Voie Mazas, 75012 Paris, France; 2 UMR 7206 Ecoanthropology, MNHN, CNRS, UP, Musée de l’Homme, 17 place du Trocadéro, 75016 Paris, France; 3 EA 2496, UP, Faculté de Chirurgie Dentaire, 1 rue Maurice Arnoux, 92120 Montrouge, France

**Keywords:** Anthropology, Evolutionary developmental biology

## Abstract

Patterns of human growth established for one population have rarely been tested in other populations. In a previous study, three growth curves from puberty were modelled for each sex in a longitudinal study of a Caucasian population based on stature, age at peak of growth and biological maturation. Each curve represents the canalisation of growth associated with the type of puberty. The high precision (± 3 cm) of individual adult stature predictions shows that growth kinetics are already set up at puberty and are canalised depending on biological maturity. Our aim is to assess whether this model can be extrapolated to other populations to test whether growth canalisation is a population-dependent phenomenon or if the model reflects a canalisation pattern specific to our species. The modelled curves predicted adult stature with the same high degree of precision in basketball players and the Baka pygmies. Therefore, (1) the relationship between growth kinetics and age at maturity is similar in all populations and (2) growth according to pubertal stages follows the same canalisation patterns in the populations despite the wide differences in their average adult statures. It suggests that morphological diversity in modern humans results from processes taking place in early development.

## Introduction

Among primates, humans are distinctive in two main respects, their polymorphism and their very particular growth pattern in absolute and relative terms, which defines what is called the ‘human life-cycle’. Despite differences in size and shape among modern human populations, human growth characterises by the presence of a childhood phase, the long lap of time between birth and age at first reproduction and relatively long brain development^1^. Maturity in Humans takes twice as long as in chimpanzees, and present accelerated growth at puberty that is not observed in our closest relative. Thus, the two main and distinctive traits of the human life cycle are the long period of growth (closely linked to the long human lifespan) and the presence of a growth spurt in adolescence^1^. These aspects have been observed in standard populations as well as in populations at the extremes of morphological variation, such as Pygmies^2^. In other words, despite the polymorphism of our species, there are aspects of growth and development that are similar between populations. The distinction between factors that are constant within our species and aspects that vary between populations allows us (1) to better understand what characterises our species compared to other species and (2) to establish the aspects that change according to the adaptation of different populations to the constraints in their environment.

The characterisation of growth in particular populations is therefore of particular interest from the evolutionary point of view. Many studies have been devoted to characterising the growth of different populations. Longitudinal studies of growth have used two methodologies, one based on the relationship between the increase in anthropometric characteristics (e.g. stature and weight) according to chronological age and the other relative to the biological maturity of individuals. In the latter, pubertal stages are defined from the age at the peak of growth and individuals are grouped into three categories according to whether secondary sexual characters show early, standard or late puberty according to specific sample^3–10^. It is important to note that the degree of biological maturity is accompanied by different growth kinetics, so that children with early or late puberty have different kinetics to children with standard puberty^11,12^. The correlation observed between adult stature and stature in early adolescence has introduced the concept of growth canalisation^13–15^.

Studies on growth canalisation are based on and widely applied to studies of so-called standard Caucasian populations e.g.^16–19^, but models have rarely been tested on populations with a different adult morphology. It has not been assessed whether growth canalisation established for one population can be extrapolated to track growth in another population, in other words, whether growth channels are population-dependent or whether the canalisation process reflects a larger phenomenon specific to our species, which can be modelled but is independent of phenotypic variations between populations.

In a previous study^20^, we modelled growth curves based on age and biological maturation in a so-called standard (Caucasian) population, from longitudinal data on stature, chronological age, age of peak of growth and secondary pubertal stages (SI, Tables S1–S5). Three mean height growth curves were proposed for each sex based on late, standard or early puberty. Each mean curve represents the growth channels of the 50th percentile of stature growth associated with each type of puberty. These curves allowed us to predict adult stature from the age of 13.3 years in boys and 10–14 years in girls. The high precision (± 3 cm) of adult stature prediction at the individual level reveals growth kinetics that are already set up at puberty and follow growth channels that depend on biological maturity.

The aim of this study is to evaluate the ability of the model established from the Caucasian population to predict the adult stature of two distinct populations with very different average adult statures at the extremes of modern human variation: a group of basketball players and a group of Baka Pygmies from Cameroon. If the model does predict adult stature in both populations, this would mean that (1) age at maturity plays a key role in or is closely related to the type of growth kinetics the individual is following and (2) growth canalisation follows the same pathways, both aspects being independent of the average adult stature of populations.

## Results

The stature and pubertal stages between 160 and 164 months of age in males, and the stature and age at menarche in female between 159 and 178 months are given in Table 1. The results for age and stature at peak growth in the Baka are given in Table 2. The percentage of males and females in both groups based on pubertal maturation is shown in Table 3. Among the Baka males, 71.4% have late-onset puberty with growth peaking at 170 to 182 months. This percentage is significantly higher than in the male basketball players (13.2%). Similarly, 42.3% of the Baka female have late-onset puberty with growth peaking between 165 and 177 months. This percentage is again significantly higher than in the female basketball players (8.8%).

**Table 1:** Mean, standard deviations and range of age, stature and pubertal stage (male) or age at menarche (female) in basketball players.

	X ± SD	Range
**Males (n = 106)**		
Age (months)	162 ± 1.2	160–164
Stature (cm)	180.2 ± 9.1	156–199
Pubertal stage	2.3 ± 0.7	1–3
**Females (n = 80)**		
Age (months)	171.1 ± 4.8	159–178
Stature (cm)	176.5 ± 7.3	161–191
Menarche age	146 ± 15.3	116–180

**Table 2:** Mean, standard deviations and range of age and stature at peak of growth in the Baka.

	X ± SD	Range
**Males (n = 14)**		
Age (months)	175.6 ± 10.3	156–185
Stature (cm)	138.8 ± 5.6	129–152
**Females (n = 26)**		
Age (months)	157.9 ± 14.9	130–180
Stature (cm)	134.4 ± 6.1	124–150

**Table 3:** Percentage distribution of biological maturation in basketball players and Baka.

Biological	Basketball players		Baka pygmies	
Maturation	Male	Female	Male	Female
Late puberty	13.2	8.8	71.4	42.3
Standard puberty	43.4	57.5	14.3	42.3
Early puberty	43.4	33.7	14.3	15.4

**Figure 1:**
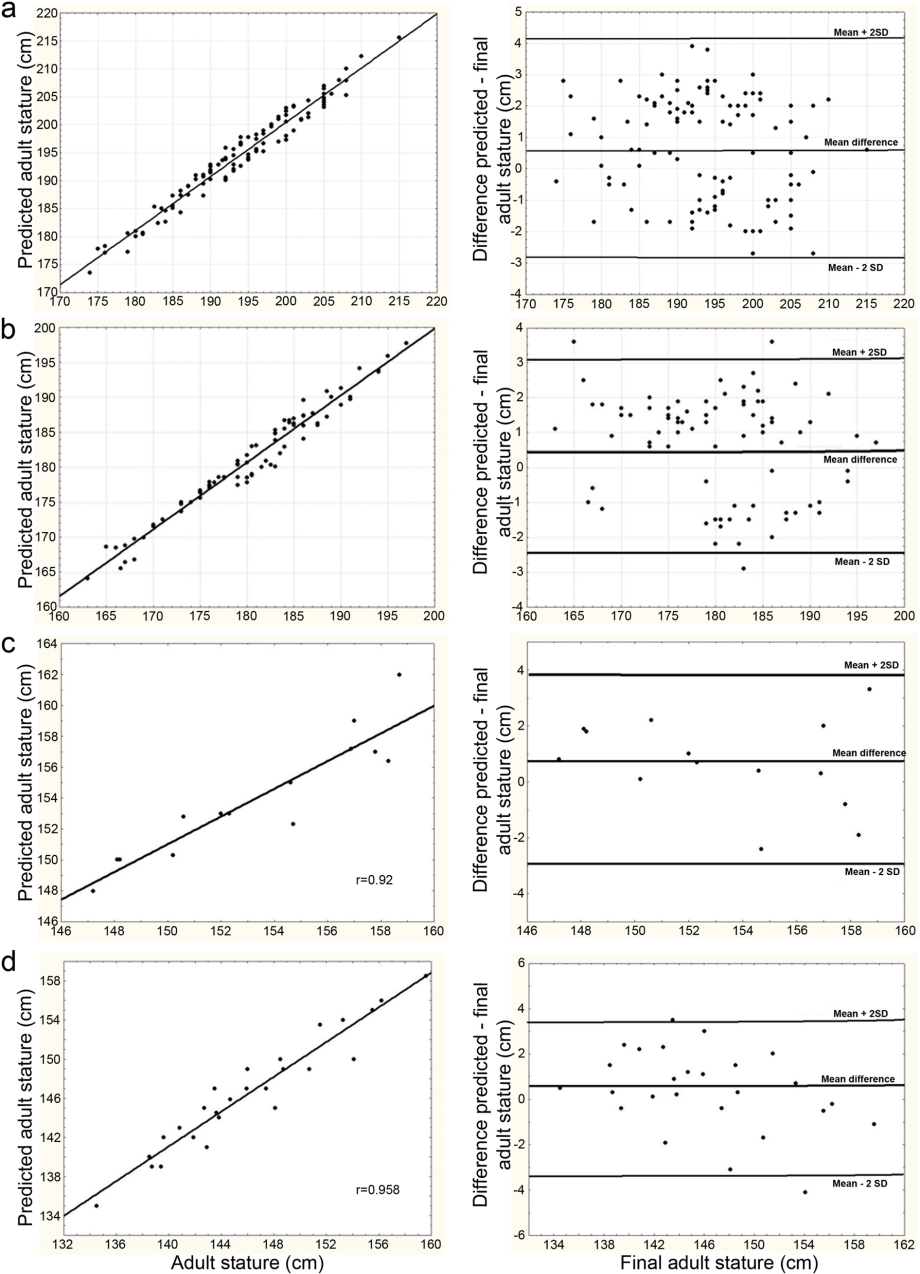
Regression curve and individual differences between estimated and actual adult stature in male (**a**) and female (**b**) basketball players and male (**c**) and female (**d**) Baka.

The values of the correlation coefficient between estimated and actual stature are very high for the two populations studied (Table 4). In addition, the mean and standard deviations of the difference between estimated adult stature and actual stature are relatively small, with a difference of 3 cm for almost all individuals (Tables 5, 6, 7) (Fig. 1).

**Table 4:** Correlation and standard error between actual and estimated adult stature.

Groups	Sex	r	SE	Δ ± sd	± 3 cm
Basketball	Male	0.98	1.7	0.7 ± 1.7	90%
Players	Female	0.98	1.7	1.7 ± 2.7	89%
Baka	Male	0.92	1.6	0.7 ± 1.6	95%
	Female	0.96	1.7	0.4 ± 1.8	92%

Average values (**Δ**) and standard deviation between actual and estimated adult stature and proportion of individuals for whom the difference between actual and estimated adult stature is less than 3 cm.

**Table 5:** Actual and estimated adult stature by maturity stages in male basketball players.

Stature	Late puberty	Standard puberty	Early puberty	Total
	n = 14	n = 46	n = 46	n = 106
Estimated	196.9 ± 10.1	192.7 ± 7.9	193.7 ± 8.5	193.7 ± 8.5
Actual	197.8 ± 10.4	193.7 ± 7.9	193.9 ± 8.2	194.3 ± 8.4

## Discussion

Many models have been proposed to predict adult stature^21–23^. Sperlich et al.^24^ performed a stature prediction for boys affected by untreated constitutional stunting using bone age methods. The prediction for 16 out of the 49 boys tested (32.6%) deviates from the final stature by more than 5 cm. Ostojic^25^ established a model for predicting the stature of young Caucasian boys practising basketball, football, volleyball and swimming using the Tanner Whitehouse method based on hand X-rays. This method resulted in a prediction of adult stature within -5.8 cm and + 4.5 cm in 95% of cases. Ali et al.^26^ developed a model for predicting stature from curvilinear regression without exposing individuals to X-rays. Their method includes age at peak growth and is therefore only applicable beyond 12 years of age for females and 14 years for males. They obtained a prediction within − 3.3 and + 5 cm with a 90% confidence interval. Lee et al.^27^ made a fairly accurate prediction of adult stature from a multiple regression that includes biological parameters. However, they introduced into the prediction equation the age immediately preceding the age at the peak of growth on the growth curve. For this reason, this method is only applicable at the beginning of the pubertal period. Sherar et al.^28^ validated and demonstrated how adult stature can be predicted using reference values obtained from sex-specific and cumulative velocity curves. Individuals were classified as having late, standard, or early maturation based on their age at the peak of growth. This method gives a rather inaccurate prediction of the stature as adults at ± 5.3 cm in 95% of cases observed in 224 males and ± 6.81 cm in 95% of cases in 120 females. The non-invasive method of predicting stature based on skeletal age, suggested by Beunen et al.^29^, makes it possible to differentiate the maturation of individuals. The mean deviation of the predicted stature is 2.3 cm with a standard estimation error of 4.7 cm at the age of 13 years. More recently, Beunen et al.^30^ proposed a model for predicting the adult stature of females without using skeletal maturation. Adult height is estimated based on stature at age 13 or 14, leg length, sitting height, and age at menarche. In 95% of cases, the prediction is in the range of ± 5.4 cm at 13 years and ± 4.6 cm at 14 years. Our previous reference study^20^ showed that associating biological maturation with chronological age makes it possible to differentiate three groups of individuals according to pubertal stages: early, standard and late puberty. These three distinct maturation groups were used to model new mean growth kinetics curves, all three of which converge to 177 cm in males and 165 cm in females, suggesting that the final average stature reached does not depend on the different stages of puberty^31^. These growth curves describing canalisation have allowed individual growth in males and females to be monitored and show high efficiency in predicting adult stature. With previous models, the difference between actual and predicted adult stature is greater than 3 cm in 95% of cases, whereas our model predictions are within ± 3 cm of the actual adult stature in 95% of cases, and are therefore more accurate.

In previous studies, models to predict adult size were obtained from industrialised populations and eventually applied to other populations whose morphology is close to that used to establish it. They have never been applied to populations with a very different adult morphology. In our study, we applied the model established from the study of a European population to two populations whose morphology lies at the extremes of current human variation, a group of basketball players and a population of Baka pygmies. The Baka pygmies is a seminomadic population of hunter-gatherers. Although the sample size in our study is limited, having 40 hunter-gatherer individuals monitored for longitudinal growth from adolescence to the adult stage based on a precise chronology is a unique case. The accuracy in the prediction of adult stature for these two populations was surprisingly similar to that for the original population, at ± 3 cm. This means that (1) the relationship between growth kinetics and age at maturity (around 11–15 years in boys and around 10–14 years in girls) is similar in all three populations and (2) that the growth depending on pubertal stages follows the same channels in all three populations despite the very wide difference in average adult stature between the Baka pygmies (153.7 cm) and the basketball players (197.8 cm). The accuracy of the model in predicting adult stature suggests that adult stature is determined as from puberty, that the stature differences between the populations at puberty (Tables 5, 6, 7) are responsible for the diversity of adult morphology and that this diverse adult morphology results from different process taking place before puberty. Two relationships can thus be distinguished. One is the relationship between biological maturity and stature at the onset of puberty, which would then be specific to each population and related to adult morphology and polymorphism. The other relationship is the link between growth kinetics at puberty and adult stature, resulting in growth canalizations that appear to be the same in all populations and would thus represent a characteristic of our species.

The applicability of our model to populations with different adult statures means that the stature difference is already present at puberty. It may be suggested that the relationship between biological maturity and stature at puberty is specific to each population and may differ between populations from the adaptation of each one to its environment. This implies that the changes in growth in relation to environmental adaptation had to take place before puberty; any change after this stage would result in a change in the relationship between pubertal growth kinetics and adult stature, and the hypothesis of similar canalisations in different populations would therefore have limitations. Few studies have been done on growth processes related to the morphological adaptation of a given population to its environment. Although the morphology of Inuit, Nilotic (Nuer. Dinka. Turkana) and of other groups with a small average adult stature has often been interpreted as adaptations to the environment, the growth processes that accompany morphology have unfortunately been very little explored.

The phenotype of Pygmies and other groups outside Africa with a small average stature and living in a forest environment has been interpreted as an adaptation to life in the forest^32^. In a study on growth in the Aeta (Philippines), Migliano et al.^33^ proposed that their small adult stature could be due to an early cessation of growth due to high mortality. If these results are confirmed, they would suggest that the small stature of the Aeta is not in itself an adaptation to life in the forest but a by-product of high mortality. The particularity in the Aeta would then be an early arrest of growth to ensure reproduction in a high risk environment. Since it occurs after the pubertal stage and thus probably without affecting age and stature at puberty, the hypothesis of a relationship common to all populations between growth kinetics at puberty and adult stature cannot be upheld and the possibility of extrapolating the model would be seriously limited. However, the lack of calibration of individual growth with chronology and some flaws in the statistical methods and interpretation of results cast serious doubts on the relevance of these conclusions^34,35^. In their study on Pume foragers, Kramer and Graves^36^ suggest that girls reach a higher proportion of their adult stature at puberty and that growth follows at low velocity in adolescence, and even after their first pregnancy, to reach a standard stature. They established that 93% of adult stature is already acquired at menarche. However, Walker et al.^37^ have estimated that 77 to 92% of adult stature is reached by 10 years of age in several forager and horticulturalist populations, so that 93% of adult stature at 12.5 years of age in the Pume does not seem to be of particular note. Furthermore, in basketball players, the proportion of adult stature at menarche varies from 97.3% in females with late-onset puberty to 99.1% in females with early-onset puberty (Tables 1, 6). In the Baka, stature at the peak of growth represents 91.9% of adult stature (Tables 2, 7), and since the menarche occurs later in the Baka, at 14.5 years of age^2^, the proportion of adult stature at menarche could be higher. To summarise, growth from puberty to adulthood in the Pume does not seem to present any particularity that would rule out a relationship between growth kinetics at puberty and adult stature. Other studies on groups with a small adult stature have not focused on a specific period of growth and only propose more general insights suggesting a general slowdown in growth^37,38^. In contrast, a longitudinal study of approximately 550 individuals of known age^2^ determined that the small adult stature of the Baka pygmies was the result of a low growth rate in early childhood. The follow-up study that Bayley^39^ carried out on the Ituri (Dem. Rep. of Congo) on births among Efe pygmies suggested that the Efe already have a small stature at birth. The pygmy phenotype therefore expresses a changing growth pattern well before puberty.

**Table 6:** Actual and estimated adult stature by maturity stages in female basketball players.

Age at menarche		Late puberty	Standard puberty	Early puberty	Total
		≥ 168	141—167	≤ 140 months	
Stature	n	7	46	27	80
	Estimated	183.5 ± 81	181.1 ± 73	179.7 ± 77	180.8 ± 75
	Actual	181.4 ± 82	179.5 ± 73	178.1 ± 76	179.2 ± 75

**Table 7:** Actual and estimated adult stature by maturity stages in the Baka pygmies.

Stature	Late puberty	Standard puberty	Early puberty	Total
Male	n = 10	n = 2	n = 2	n = 14
Estimated	154.7 ± 4.6	152.7 ± 0.5	151.7 ± 1.9	154 ± 4
Actual	153.7 ± 4.7	153.4 ± 1.9	151.3 ± 1.5	153.3 ± 4.1
Female	n = 11	n = 10	n = 5	n = 26
Estimated	146.8 ± 7.3	147.3 ± 5	144.6 ± 4	146.6 ± 5.8
Actual	146.6 ± 7.6	146.7 ± 5.6	144.1 ± 4.7	146.2 ± 6.3

The idea that changes during the developmental period before puberty can ensure adaptation of a population to a particular environment is broadly in agreement with the concept of predictive adaptive responses^40^. This concept suggests that in mammals, irreversible developmental pathways are taken early in life in order to cope with environmental conditions and maximise reproductive fitness as adults. This concept mainly concerns processes occurring in the embryonic, foetal and perinatal periods and corresponds to the processes described for Pygmies. What is certain is that the earlier these changes take place during development, the less energy is required to produce them. Our results lead us to suggest that growth from puberty follows canalisation patterns that are shared by populations with very different adult morphologies and that each population is characterised by a particular relationship between stature and age at biological maturity. If so, the morphological diversity of our species would result from processes that take place in early development, no later than childhood, most probably as adaptive responses to environmental constraints.

## Materials and methods

The growth canalisation established in the study of a Caucasian population made it possible to predict the adult stature of each individual with a very small discrepancy (± 3 cm) (SI). If the result is also satisfactory for other populations with different average adult statures, this means that the kinetics are the same and therefore do not depend on the population analysed: they are independent of the diversity of adult morphology among populations and canalisations can therefore be extrapolated.

**Figure 2:**
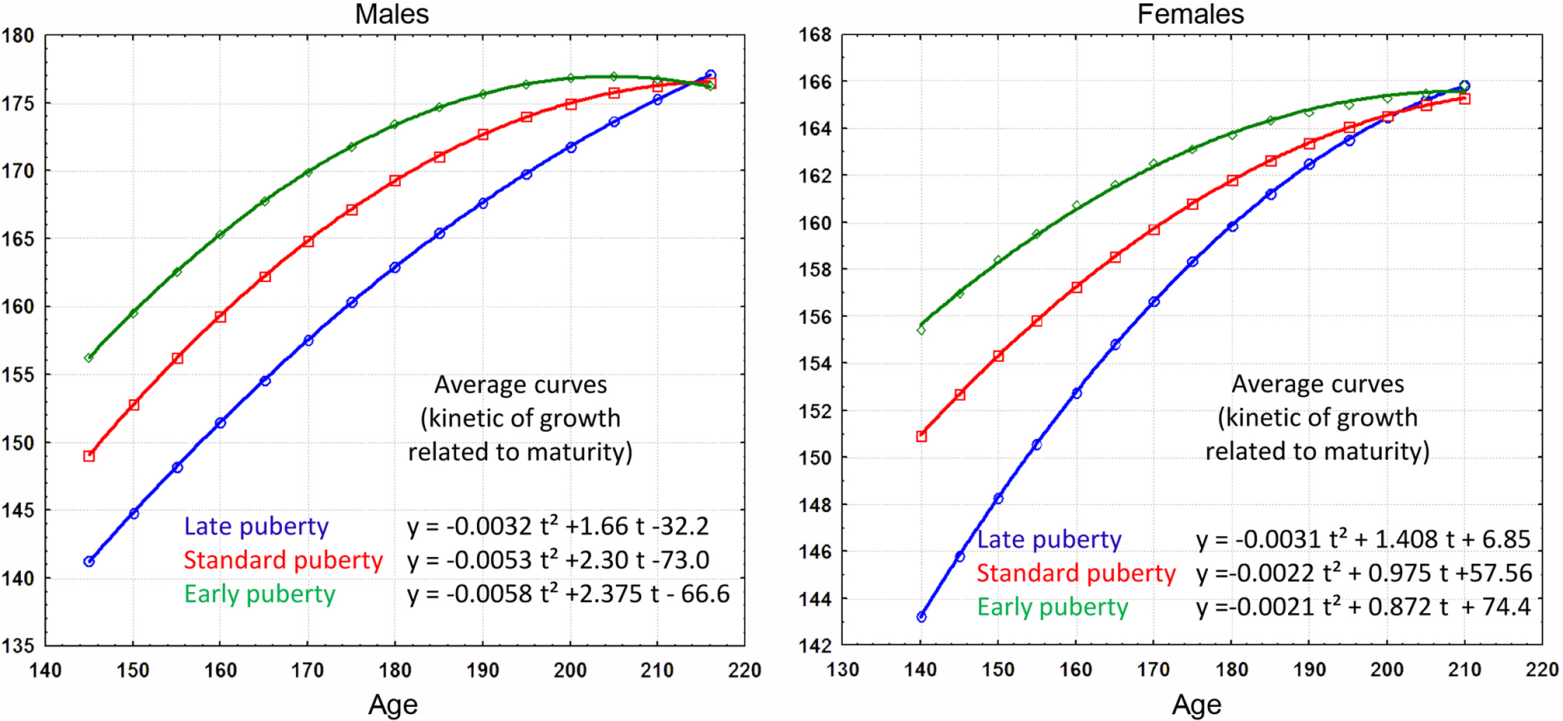
Polynomial curves of degree 2 in stature (y = cm) for males and females according to age (t = months). These curves were modelled over the age range of 120 to 216 months using average curves that represent growth kinetics as a function of biological maturation. In the projection, average stature values converge at around 177 cm at the age of 216 months (18 years) for males and around 165 cm at the age of 17.5 years for females. The standard deviation for stature is 6 cm in males and 5.6 cm in females.

To test whether the growth kinetics established from a standard reference population can be extrapolated to other populations with different adult morphologies, the mean growth curves obtained for the reference population (Fig. 2) were used to predict the adult stature of individuals in two populations representing the widest variation in human stature, a group of basketball players and a group of Baka pygmies. The first sample consisted of 186 adult basketball players (106 males and 80 females), the vast majority of Caucasian origin. As the study of this cohort is cross-sectional, the stature, chronological age and pubertal stages between the ages of 160 and 164 months were identified for boys. The stature of the girls was measured between 159 and 178 months and the age at menarche was obtained by questionnaires. The actual adult stature of these individuals is known, with the females averaging 179.2 cm and the males 194.3 cm. These data were collected in 2013 and 2014 as part of a research project with the French Basketball Federation ("Predictaille" software). All participants and their parents gave their oral and written consent to participate in the studies in accordance with the Helsinki Declaration.

The second sample is made up of 40 individuals (26 females and 14 males) from Moange le Bosquet, a Baka pygmy village in south-eastern Cameroon. African pygmies, who include many different groups, live in equatorial rain forests and grow to an average adult stature of < 155 cm^41^. Pygmies share a common ancestor and split from Bantu-speaking populations at around 60,000 years BP^42^. The genetic bases of this very specific phenotype are polygenic. They include genes from the growth hormone axis (GH – IGFI) as well as genomic and gene regions that relate to the traits themselves^43–45^. The pygmy phenotype itself is usually interpreted as an adaptation to life in equatorial rain forests^32^.

The Baka pygmies have an average adult stature of 146.7 cm (sd = 4.7) in females and 153.5 cm (sd = 6.2) in males^2^. Birth records had been kept for many years in Moange le Bosquet by nuns in a medical centre run by the catholic mission. They are available from 1980 to 1983 and from December 1987 to the present. We conducted one field study each year in May–June from 2007 to 2017, and an additional field study in October from 2011 to 2015. We systematically measured the stature and weight of Baka individuals of known age (3–25 years of age) living in the locality. Thanks to the birth records, we were able to describe growth and life history variables based on an absolute chronology^2,35,46^. The individuals included in the present study are a subset of those included in the previous one. We must remember that the Baka pygmies are semi-nomadic hunter-gatherers and although our field works were carried out systematically, performing the same measurements on the same individuals for many years is a very difficult task as either the individuals are not present in the locality at the time of our stay, they have established residence in a camp in the middle of the forest or in another locality. The stature of the 40 individuals included in our study was measured systematically for 8–10 years from adolescence to adulthood (from 12 to 19 years of age). Although this is a limited sample, it represents the only case of hunter-gatherers in which a longitudinal study could be conducted by taking measurements at adolescence and adult based on an absolute chronological calibration thanks to birth records (also unique case among hunter-gatherers). The secondary sex characteristics of the males and the age at menarche of the females were not recorded, but the age at peak growth in stature was determined from longitudinal data, making it possible to identify whether biological maturation was late, standard or early according to the criteria set out in Tables S2 and S3.

Prior informed consent was obtained from all participants and from both parents of any participants aged under 18. All methods are non-invasive and were carried out in accordance with relevant guidelines and regulations. The study obtained approval from the French Centre National de la Recherche Scientifique (CNRS), Agence National de la Recherche (ANR) and the Institut de Recherche pour le Développement (IRD) and was carried out under an international agreement between the IRD and the Cameroon Ministry of Scientific Research and Technology.

The association between maturation stage and chronological age allowed mean growth kinetics curves to be built that showed a high degree of accuracy in predicting adult stature in the baseline study (SI). By applying the growth kinetics curves and the projection of the Z-score obtained with the reference sample, we proposed a prediction of adult (18-year-old) stature in both cohorts. The first step was to determine whether puberty was late, standard or early in each individual. The Z score for stature was then calculated from the age and mean stature value on the corresponding mean curve as a function of biological maturity (Fig. 1, Tables S4, S5). Finally, a projection of the Z score was made to estimate stature at the age of 18. For example, one boy Baka reached his growth peak at 162 months (13.5 years) and measured 125.4 cm. He thus had a late-onset puberty and his growth kinetics are represented by the curve y = − 0.0032 t^2^ + 1.66 t – 32.2 (Fig. 1). On this curve, the average stature at 162 months is 152.7 cm. Thus, Z = (125.4–152.7)/6 = − 4.55, where 6 is the standard deviation for males. The projection of his stature to 18 years of age (216 months) is 177 + 6 * (− 4.55) = 149.7 cm. His actual stature at 18 years of age is 148.1 cm, a difference of 1.8 cm.

The individual differences between the resulting estimates and the actual adult stature values were compared and the specified r^2^ values and standard estimation errors (SEE) obtained. The values of the lower and upper limits within a 95% confidence interval on either side of the mean deviation are given. For all tests, α = 0.05. The statistical analysis was carried out using Statistica software (version 6, Tulsa, Olka, USA). If the prediction of adult stature in basketball players and the Baka pygmies is effective, the growth kinetics and stature at the beginning of puberty, always taking biological maturity into account, will be similar for all populations and will follow the same canalisation pattern regardless of adult morphology.

## Supplementary information

Supplementary information.

## Data Availability

The datasets generated during and/or analysed during the current study are available from the corresponding author on reasonable request.
