# Supplementary material for: The same growth pattern from puberty suggests that modern human diversity results from changes during pre-pubertal development
Source: Sci Rep. 2021 Mar 1;11:4817. doi: 10.1038/s41598-021-84327-1 (PMC7921106; doi:10.1038/s41598-021-84327-1)
Supplement: Supplementary file 1 — Supplementary information. [file 41598_2021_84327_MOESM1_ESM.pdf]

The same growth pattern from puberty suggests that modern human diversity results from changes during pre-pubertal development.

Jean-Claude Pineau, Fernando V. Ramirez Rozzi.

Supplementary Information

We conducted a longitudinal study of growth in stature based on chronological age and biological maturation [20]. Longitudinal data were collected twice a year over five consecutive years from a cohort of 125 boys aged 12-17.5 years and 154 girls aged 11-17 years. The data were collected in three schools in the Soissons region in the Aisne department (northern France). The students were from three different socio-professional groups: workers, skilled workers and middle managers. The sample can be considered very homogeneous since 98% were of Caucasian origin. All participants and parents gave their oral and written consent. Authorisation from the Aisne Medical Council and Regional Education Authority was also obtained in accordance with the Helsinki Declaration.

The data obtained are for stature and chronological age in both sexes. Stature was measured in the standard standing position by the same operator throughout the study. The exclusion criterion concerned pupils whose stature-for-age Z score was lower or higher than 3 standard deviations. In the boys, an assessment of secondary pubertal stages was also performed using a simplified version of the Tanner stages. Pubertal stages were determined from facial hair, axillary hair and voice change. The degree of maturation was thus observed from four distinct stages: stage ST1 at the beginning of puberty (Tanner Stage 0); pre-puberty stage ST2 (Tanner stages 1 and 2); para-puberty stage ST3 (Tanner stage 3) and puberty stage ST4 (Tanner stages 4 and 5). When there was a match between the facial hair and the axillary hair, the stage indicated was used. If there was a gap between the two stages the later stage was selected. In practice, the majority of observed stages do not differ by more than two consecutive stages (Table S1). These secondary pubertal stages associated with chronological age allowed us to differentiate biological maturity between the boys. In fact, in the 160 to 164 month age range, the maturation/age association allowed us to differentiate between boys with early, standard and late puberty with no possibility for intersection and to obtain the average age at the peak of growth (Table S2).

In girls, the biological maturity assessment was based on the age of onset of the first menstrual period (age at menarche), which was the only criterion for puberty needed for this

study. There is a significant relationship between the age at menarche and the age at the peak of growth (r=0.94) [47]. We observed a close association between age at peak growth and age at menarche and established 3 distinct puberty groups (early, standard and late) whose extremes of age at peak growth do not intersect (Table S3).

We thus differentiated individuals by age at peak growth as shown in Tables S2 and S3. Age at peak growth in stature was thus the determining criterion for developing new average growth curves. Three mean age-related curves for stature were then modelled for each group of boys and girls based on biological maturation (early, standard and late puberty) (Figure 2). Each curve therefore represents the growth kinetics as a function of biological maturation. The mean growth curves were applied to the reference population to test the degree of accuracy of adult stature prediction. In 95% of cases the estimated stature was within 3 cm of the actual stature, so the predictive value is very high.

To estimate the adult stature of individuals based on knowledge of chronological age, stature and biological maturation (pubertal stages in boys and age at menarche in girls), it was first determined whether puberty was late, standard or early. The Z score for stature was then calculated from the subject's age, and the mean stature value on the corresponding mean curve as a function of biological maturity (Figure S1 Tables S4 S5). Finally a projection of the Z score was made to estimate stature at the age of 18. To take the example of, a boy measuring 178 cm at the age of 163 months: if the boy's puberty was late, his Z score will be equal to: (178-153.4)/6 = 4.1 cm where 6 is the standard deviation in boys. Therefore at the adult stage (at 216 months = 18 years) his stature will be: 177cm + 6 x 4.1cm = 201.6 cm, where 177 cm is the average stature obtained in the modelling (Figure S1). If the boy has a standard puberty Z score = (178-161.1)/6 = 2.81 cm; and at 216 months his stature will be 177cm + 6 x 2.81cm = 193.9 cm. If the boy has an early puberty Z score = (178-166.8)/6 = 1.87 cm; and at 216 months his stature will be 177cm + 6 x 1.87cm = 188.2 cm.

The association between maturity and chronological age has proved to be a powerful biological criterion for predicting stature in adulthood from the age of 13 without waiting for the peak of growth.

References

20 Pineau, J. C. Prediction of male basketball players' adult stature from the age of 13 years using chronological age and maturity. *J. Sport Med. & Phys. Fitness* **59**, 613-618 (2019). Doi: 10.23736/S0022-4707.18.08784-4

47. Pineau, J. C. Etude de la croissance longitudinale de la stature d'adolescentes en fonction de l'âge et de la puberté. *Antropo* **38**, 39-45 (2017).

Supplementary Tables

Table S1: Pubertal Stages in Boys

Tanner Stage	ST1<br>Before puberty<br>Stage 0	ST2<br>Pre-pubescent<br>Stages 1 and 2	ST3<br>Para-pubescent<br>Stage 3	ST4<br>Pubescent<br>Stages 4 and 5
Axillary hair	No hair	Start of hair growth	Very visible	Thick hair growth
Facial hair	No facial hair	Facial hair	Moustache	Abundant beard hair
Voice change	Soft voice	Voice instability	Deep voice	Deep voice

Table S2: Age at peak growth in boys as a function of biological maturation.

	Age (months) at peak of growth		Pubertal stages
Puberty	*Mean*	*[range]*	
Late	177.2	[170-182]	A1
Standard	166.4	[161-169]	A2
Early	155.4	[146-160]	A3

Table S3: Age at peak growth and age at menarche in females based on biological maturation.

	Age (months)			
	peak of growth		menarche	
Puberty	*Mean*	*[range]*	*Mean*	*[range]*
Late (n=26)	169.5	[165-177]	171.3	[168-180]
Standard (n=98)	152.4	[141-164]	152.1	[141-167]
Early (n=30)	135.3	[125-140]	133.9	[123-140]

Table S4: Mean stature for late, standard and early puberty between 160 and 164 months for boys in the reference population.

STATURE (cm)	AGE (months)					
	Puberty	*160*	*161*	*162*	*163*	*164*
	Late	151.5	152.1	152.7	153.4	154.0
	Standard	159.3	159.9	160.5	161.1	161.7
	Early	165.3	165.8	166.3	166.8	167.3

Table S5: Mean stature at late, standard and early puberty between 120 and 170 months in females in the reference population.

STATURE (cm)	AGE (months)											
	Puberty	*120*	*125*	*130*	*135*	*140*	*145*	*150*	*155*	*160*	*165*	*170*
	Late	131.2	134.4	137.5	140.4	143.2	145.8	148.3	150.6	152.8	154.8	156.6
	Standard	142.9	145.1	147.1	149.1	150.9	152.7	154.3	155.8	157.2	158.5	159.7
	Early	148.8	150.6	152.3	153.8	155.3	156.7	158.0	159.1	160.2	161.1	162.0
